# Longitudinal multi-omics transition associated with fatality in critically ill COVID-19 patients

**DOI:** 10.1186/s40635-021-00373-z

**Published:** 2021-03-15

**Authors:** Chaoyang Sun, Yuzhe Sun, Ping Wu, Wencheng Ding, Shiyou Wang, Jiafeng Li, Langchao Liang, Chaochao Chai, Yu Fu, Zhiming Li, Linnan Zhu, Jia Ju, Xin Liao, Xiaoyuan Huang, Ling Feng, Ding Ma, Liang He, Dongsheng Chen, Gang Chen, Xin Jin, Peng Wu

**Affiliations:** 1 Cancer Biology Research Center (Key Laboratory of the Ministry of Education), Tongji Medical College, Tongji Hospital, Huazhong University of Science and Technology, Wuhan, China; 2 Department of Gynecologic Oncology, Tongji Hospital, Tongji Medical College, Huazhong University of Science and Technology, Wuhan, China; 3 BGI-Shenzhen, Shenzhen, 518083 China; 4 BGI Education Center, University of Chinese Academy of Sciences, Shenzhen, 518083 China; 5 Shenzhen Bay Laboratory, Shenzhen, 518107 China; 6 Department of Geriatrics, Tongji Hospital, Tongji Medical College, Huazhong University of Science and Technology, Wuhan, Hubei Province China; 7 Department of Gynecology and Obstetrics, Tongji Hospital, Tongji Medical College, Huazhong University of Science & Technology, Wuhan, China; 8 School of Medicine, South China University of Technology, Guangzhou, 510006 Guangdong China; 9 Guangdong Provincial Key Laboratory of Human Disease Genomics, Shenzhen Key Laboratory of Genomics, BGI-Shenzhen, Shenzhen, 518083 China

**Keywords:** COVID-19, Multi-omics, Critically ill, Erythrocyte, Melatonin

## Abstract

**Purpose:**

Critically ill COVID-19 patients have significantly increased risk of death. Although several circulating biomarkers are thought to be related to COVID-19 severity, few studies have focused on the characteristics of critically ill patients with different outcomes. The objective of this study was to perform a longitudinal investigation of the potential mechanisms affecting the prognosis of critically ill COVID-19 patients.

**Methods:**

In addition to clinical data, 113 whole blood samples and 85 serum samples were collected from 33 severe and critical COVID-19 patients without selected comorbidities. Multi-omics analysis was then performed using longitudinal samples.

**Results:**

Obvious transcriptional transitions were more frequent in critical survivors than in critical non-survivors, indicating that phase transition may be related to survival. Based on analysis of differentially expressed genes during transition, the erythrocyte differentiation pathway was significantly enriched. Furthermore, clinical data indicated that red blood cell counts showed greater fluctuation in survivors than in non-survivors. Moreover, declining red blood cell counts and hemoglobin levels were validated as prognostic markers of poor outcome in an independent cohort of 114 critical COVID-19 patients. Protein–metabolite–lipid network analysis indicated that tryptophan metabolism and melatonin may contribute to molecular transitions in critical COVID-19 patients with different outcomes.

**Conclusions:**

This study systematically and comprehensively depicted the longitudinal hallmarks of critical COVID-19 patients and indicated that multi-omics transition may impact the prognosis.

**Take home message:**

Frequent transcriptional phase transitions may contribute to outcome in critically ill COVID-19 patients. Furthermore, fluctuation in red blood cell and hemoglobin levels may relate to poor prognosis. The biological function of melatonin was suppressed in COVID-19 non-survivors, which may provide a potential theoretical basis for clinical administration.

## Introduction

Coronavirus disease 2019 (COVID-19) was declared a global pandemic by the World Health Organization (WHO) on March 11, 2020. Despite an overall 2% mortality rate in COVID-19 patients, 53.8–61.5% of critically ill patients deceased within 28 days of admission to intensive care units (ICU) [1–3]. Thus, there is an urgent medical need for the overall assessment of patients’ condition and early intervention for these high-fatality cases.

Time-series sampling of patients can capture the dynamic nature of disease over time and highlight the biological changes that occur as disease progresses. Previous longitudinal studies of COVID-19 have primarily focused on the immune response against pathogens [4, 5]. At present, sequencing-based multiscale changes among critical COVID-19 patients and their potential correlation with clinical trajectory remain unknown. Exploration of these molecular changes could contribute to our understanding of COVID-19 and help uncover potential therapeutic targets. Many studies have shown that comorbidities impact the COVID-19 prognosis [6, 7]. Therefore, we studied patients without selected comorbidities (see Methods).

In this study, we acquired longitudinal transcriptomic, lipidomic, proteomic, and metabolomic data from COVID-19 patients. Based on these multi-omics data, we identified patients' specific conditions in ICU-admitted COVID-19 patients, and significant variations were further identified during hospitalization. Core pathways related to changes in disease condition in those patients that survived were also investigated.

## Results

### Overview of study design and COVID-19 samples demographics

**Fig. 1:**
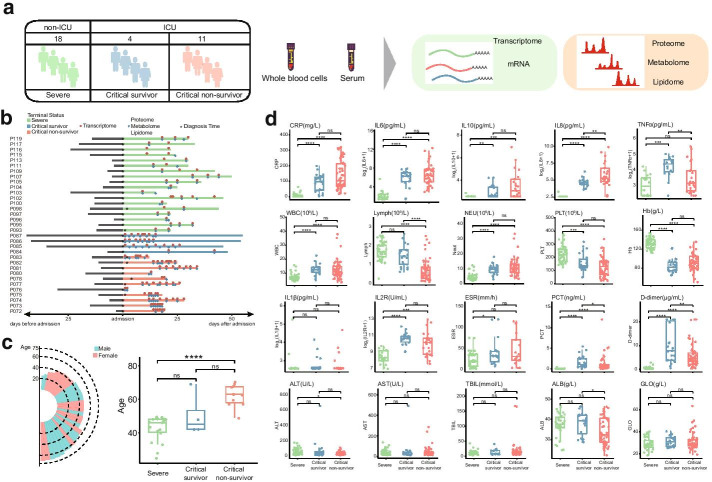
Patient enrollment, study design and different clinical parameters of COVID-19 patients. **a** Summary of recruited COVID-19 patients, including 18 severe patients, 4 critical survivors, and 11 critical non-survivors. **b** Admission days and multi-omics sample collection time of COVID-19 patients. Datasets contained 113 transcriptome, 85 proteome, 85 metabolome, 85 lipidome were collected from 33 patients. Grey bar records time from disease onset to admission by weeks; colored bar records time from admission to outcome-time-point by days. Points represent multi-omics data sampling time. The details of enrolled patients were shown in Additional file 1: Table S1. **c** The age and gender distribution and comparison of 33 COVID-19 patients. *p < 0.05. **d** The clinical parameters of COVID-19 patients. 123 clinical data were collected. ****p < 0.0001, ***p < 0.001, **p < 0.01, *p < 0.05. WBC, white blood cells; Lymph, lymphocyte; NEU, neutrophil; PLT, platelet; Hb, hemoglobin; ALT, alanine transaminase; AST, aspartate aminotransferase; TBIL, total bilirubin; ALB, albumin; GLO, globulin; CRP, C-reactive protein; IL6, interleukin-6; IL10, interleukin-10; IL8, interleukin-10; TNFα, tumor necrosis factor alpha; IL1β, interleukin-1 beta; IL2R, Interleukin-2 receptor; ESR, erythrocyte sedimentation rate; PCT, procalcitonin; D-dimer, fibrin D-dimer

To investigate the physiological and biochemical changes in COVID-19 patients, we collected longitudinal blood samples from 33 COVID-19 patients, including 18 severe patients, 11 critical non-survivors, and 4 critical survivors (Fig. 1a). In total, we assessed 113 whole blood samples and 85 serum samples collected over 2–7 longitudinal time-points from 0 to 55 days after hospital admission (Fig. 1b). There were no significant differences in age or sex between critical non-survivors and survivors, and the effects of age and sex were adjusted in our further analysis (Fig. 1c, Additional file 1: Table S1). Basic demographic information stratified by disease severity is detailed in Additional file 1: Table S1.

### Clinical parameters could not distinguish critical survivors and non-survivors

Among the 20 laboratory parameters measured, four did not show significant differences among the three groups. In contrast, 8 parameters, such as white blood cells (WBC), neutrophils (NEU), platelets (PLT), hemoglobin (Hb), C-reactive protein (CRP), interleukin-6 (IL-6), IL-10, and IL-2R, showed significant differences between the severe and critical groups. However, these differences did not exist between critical non-survivors and survivors (Fig. 1d). Therefore, we inferred that severe and critical patients could be clearly distinguished by clinical parameters, but clinical data resolution was insufficient to clarify critical patients with different outcomes.

### Multi-omics analysis identified distinct features in COVID-19 patients with different severity

We acquired proteomic, metabolomic, and lipidomic data from sequencing serum samples, and transcriptomic data from whole blood cells. Differential expression analyses of the multi-omics profiles were separately performed using multiple testing, with 2101 mRNAs, 3 proteins, 38 metabolites, and 10 lipids identified as significantly altered between severe and critical samples (Additional file 3: Figure S1). Many differentially expressed genes were observed in the transcriptomic data, suggesting that transcriptomes may be a good data source to assess patient status.

### Phase transition in transcriptome of critically ill COVID-19 patients

**Fig. 2:**
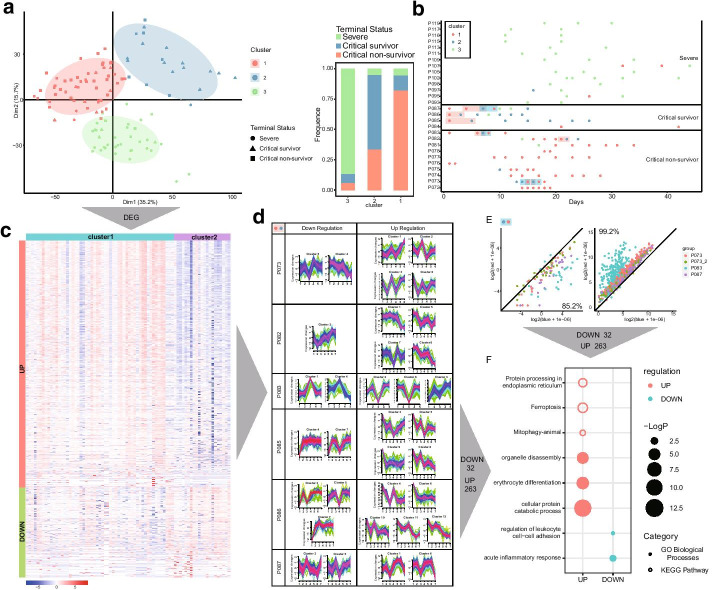
Transcriptome features of phase transition between Cluster 1 and Cluster 2. **a** Left, PCA map for transcriptomics of 113 samples; right, the percentage of terminal status in each cluster. Dots represent samples. Three colors represent three clusters. Round, triangle, and square shapes represent patients’ terminal status. **b** Longitudinal distribution of samples in colors of clusters. 113 transcriptomic data were represented at time-points. Scatter plot shows the RNA sample collection time with dots colored by clusters. Red box-shadow represents Cluster 1-to-2 transition. Blue box-shadow represents Cluster 2-to-1 transition. **c** Heatmap of differential expression genes (logFC > 1) between Cluster 1 and Cluster 2. **d** Soft clustering of longitudinal gene dynamics in six patients containing Cluster 1-to-2 transition (FDR < 0.05). Each patient is individually analyzed by using mfuzz. X-axis represents the sampling time. **e** Validation of differential expressed gene from Cluster 1-to-2 by using Cluster 2-to-1 transcriptome data. Color represents the patient id. 85.2% up-regulated genes and 99.2% down-regulated genes show reversed expression levels. **f** GO terms and KEGG pathways for differentially expressed genes between Cluster 1 and Cluster 2. Top 3 terms and pathways are displayed. The size of dots denotes the -log_10_ of the p-value, the color denotes the expression level

To assess critical patients with different clinical outcomes and better identify the key turnover in their in-hospital period, we used 113 transcriptomic profiles to perform principal component analysis (PCA) and clustering analysis. PCA roughly divided patient samples into three categories that were highly correlated with disease severity and clinical outcome (Fig. 2a). The distribution of the three clusters showed that each cluster mainly corresponded to one group of the patients (Fig. 2a). However, Cluster 2 contained approximately 30% of non-survivor samples suggesting that the longitudinal samples of critical survivors had a different gene expression pattern (Fig. 2a). We further displayed the clustering results in longitudinal order and found that most initial time-points of critical patients were classified into Cluster 1 (Fig. 2b). Interestingly, after the first Cluster 1 time-points (P085, P086 and P087), in-hospital time-points of three critical survivors transformed from Cluster 1 to Cluster 2, while overall cluster transition was much lower among critical non-survivors (Fig. 2b). These results indicate that phase transition in critical survivors, but not in non-survivors, may be an important reason for the different outcomes.

By analyzing the differential expression of genes between Cluster 1 and Cluster 2, we identified 1319 up-regulated and 478 down-regulated genes in Cluster 1 (Fig. 2c, Additional file 2: Table S2). To further explore gene expression patterns over time, we selected six patients, whose transcriptome features changed from Cluster 1 to Cluster 2 in adjacent time periods (Fig. 2b, red box-shadow). Differential genes were divided by Mfuzz software into clusters with similar patterns along longitudinal time-points. We identified differentially expressed genes in Cluster 1-to-2 transition in all six time-point pairs, including 263 up-regulated genes and 32 down-regulated genes (Fig. 2b–d). To verify the reliability of these 295 genes, we further selected 4 paired of adjacent time-points showing Cluster 2-to-1 transfer, with 85.2% of down-regulated genes and 99.2% of up-regulated genes showing consistency (Fig. 2e, blue box-shadow). Thus, the expression of 295 genes showing cluster transition may be key factors that change during the time in which patients exhibit critical disease. Enrichment analysis further showed that, compared to Cluster 2, genes related to acute inflammation response decreased in Cluster 1, while genes related to protein catabolism, erythrocyte differentiation, ferroptosis, and organelle disassembly increased (Fig. 2f). These results suggest that cellular catabolism was enhanced, but the immune response was somehow weakened in Cluster 1. As approximately 80% of Cluster 1 samples were from deceased patients, the overall physiological changes observed in Cluster 1 may highlight the biological function of these pathways in COVID-19 patient prognosis (Fig. 2a).

As we collected multiple samples at different time-points of disease progression in each patient, the differences in sample number and sampling time may impact the results. We adjusted individual (different samples from the same patient) and sampling time (sampling day from disease onset), then re-analyzed the differentially expressed genes and their enriched pathways. Up-regulated genes were enriched in protein catabolism, erythrocyte development, mitophagy, and ferroptosis, whereas down-regulated genes were related to endothelial cells (Additional file 4: Figure S2). The robustness of the up-regulated genes indicates that protein catabolism, erythrocyte development, mitophagy, and ferroptosis are likely to be major changes that occur during Cluster 1-to-2 transition. We also assessed what clinical features contributed most to gene expression variance using the Adonis test (Additional file 4: Figure S2). Individual and sampling time ranked 3 and 6 in the list, suggesting that the donor and sampling time were not the most important factors. Interestingly, Hb ranked higher than sampling time (Additional file 4: Figure S2).

### Dynamic changes in red blood cell (RBC) and Hb levels associated with prognosis in critical COVID-19 patients

**Fig. 3:**
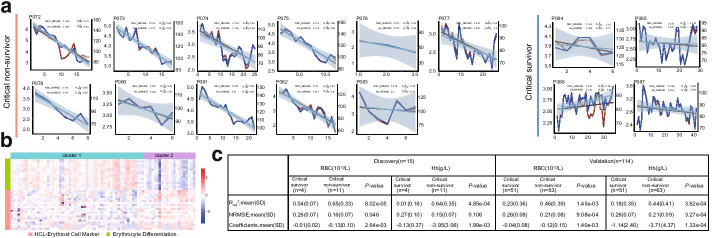
The dynamic changes of RBC and Hb levels in critically ill COVID-19 patients with different outcome. **a** Red blood cell (RBC) count and hemoglobin (Hb) of critical(survival) and critical(death) patients. The red and blue lines represent the RBC and Hb levels, respectively. Normalized root means square error (NRMSE) and *R*_adj_^2^ are marked at the top right. **b** The heatmap of erythrocyte differentiation genes and HCL-erythroid cell marker genes in Cluster 1 and Cluster 2. **c** The RBC and Hb characteristics of COVID-19 patients in discovery and validation groups

Based on transcriptomic data, the erythrocyte differentiation pathway was up-regulated, which aroused our interest in RBC. Therefore, we analyzed RBC and Hb levels in critical patients during their hospitalization. Longitudinal regression showed that RBC counts in 11 critical non-survivors gradually declined over time, and Hb values were highly correlated with RBC counts (Fig. 3a, Additional file 2: Table S2). However, RBC and Hb levels of 4 critical survivors fluctuated constantly or gradually increased. The *R*_adj_^2^ of the linear regression and normalized root mean square error (NRMSE) indicated that, compared to the critical survivors, the instability of RBC values was significantly lower in critical non-survivors (Fig. 3a). Moreover, the expression levels of erythrocyte cell markers from the human cell landscape (HCL) were higher in Cluster 2 than in Cluster 1 [8]. Although erythrocyte marker genes were highly expressed in Cluster 2, the expression of erythrocyte differentiation genes was higher in Cluster 1 (Fig. 3b). Thus, these results suggest that critical patients with different outcomes may have different trends in RBC and Hb levels.

To validate the above results, we collected RBC and Hb data from 114 independent COVID-19 critical patients without selected comorbidities (51 survivors and 63 non-survivors). Consistently, RBC and Hb levels in critical survivors showed more significant fluctuation than critical non-survivors (Fig. 3c), indicating that a progressive decline in erythrocytes may be related to prognosis in critical patients. Yet, the underlying mechanism of this potential correlation needs further investigation.

### Protein–metabolite–lipid network analysis indicated activation of tryptophan metabolism in Cluster 1

**Fig. 4:**
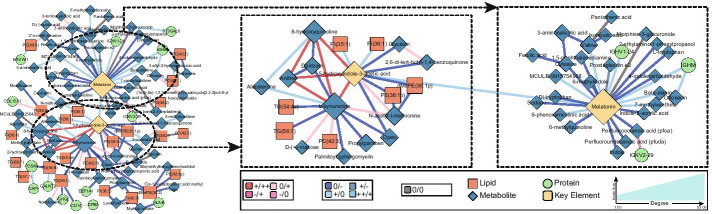
Protein–metabolite–lipid multi-omics network. The network of analytes whose correlation has been converted identically in Cluster 1 vs. Cluster 2 and critical vs. severe. Nodes are colored and shaped based on the type of molecular and the size of nodes corresponds to their degree centrality. The color of edges represents the pattern of correlation changes. Crimson line +/++ in the upper legend of the networks indicates that correlation between two connected analytes pairs was positive (+) in critical group, and the correlation became even more strongly positive (++) in severe group samples, as defined by statistically significant (p < 0.05) increase in correlation coefficients between the analytes pair across the two conditions. LightPink line 0/+: insignificant correlation in critical group → positive correlation in severe group. DeepPink line −/ + : negative correlation in critical group → positive correlation in severe group. HotPink line −/0: negative correlation in critical group → insignificant correlation in severe group. Blue line 0/−: insignificant correlation in critical group → negative correlation in severe group. SteelBlue line ± : positive in critical group → negative in severe group. LightSkyBlue line +/0: positive in critical group → insignificant in severe group. DeepSkyBlue line ++/+: more strongly positive in critical group → positive in severe group. DimGray line 0/0: insignificant correlation in critical group → insignificant correlation in severe group

The “cytokine storm” caused by the uncontrolled hyperproduction of proinflammatory cytokines and chemokines can be fatal in COVID-19 patients [2]. The altered inflammatory signaling is accompanied by a specific change in metabolites and metabolic processes [9]. Based on the relationships among proteins, metabolites, and lipids, we analyzed the protein–metabolite–lipid networks in 85 samples, with the significant changes during prognosis selected. Metabolites related to tryptophan metabolism, such as melatonin, 5-hydroxyindole-3-acetic acid, and l-kynurenine were significantly changed between Clusters 1 and 2. Melatonin, which is synthesized by a precursor provided by tryptophan metabolism, became negative regulation with multiple factors in Cluster 1 compared with Cluster 2. Furthermore, 5-hydroxyindole-3-acetic acid and l-kynurenine were positively correlated with most compounds in the network in Cluster 1 and associated with increased expression levels (Fig. 4, Additional file 5: Figure S3). Considering the essential role of tryptophan metabolism in erythrocyte differentiation, the accelerated tryptophan metabolism in Cluster 1 may contribute to the observed enrichment in the erythrocyte differentiation pathway based on the transcriptomic data [10].

## Discussion

To better understand how multi-omics features of critical COVID-19 patients change over time, we profiled integrated studies of multiple molecular factors for 15 critical COVID-19 patients and 18 severe patients as a control population. By leveraging a multi-omics view on longitudinally collected samples, we identified various characteristics between critical surviving and non-surviving COVID-19 patients.

Through gene expression, our evaluation of patient condition drafted a dynamic map over time. Coincidentally, most samples obtained from critical patients during hospital admission belonged to Cluster 1. The expression classification of three survivors (P085, P086 and P087) changed from Cluster 1 to Cluster 2 during longitudinal analysis, which may be a crucial factor contributing to their survival (Fig. 2b). Furthermore, transcriptomic analysis of temporal changes during Cluster 1-to-2 transition provided three major insights, including acute inflammation response, protein catabolism (especially autophagy and mitophagy), and ferroptosis (Fig. 2f). Immunological response was one of the most important pathways, while autophagy genes were up-regulated in Cluster 1-to-2 transition (Fig. 2f). Although critical COVID-19 patients who suffer from multiple organ dysfunction may exhibit dysregulation of many pathways, our research showed that activation of autophagy likely plays a central role in the prognosis of critical COVID-19 patients. Thus, effective therapies targeting the autophagy pathway may be promising therapeutic methods for COVID-19 patients [11]. Our analysis indicated that critical COVID-19 patients could benefit from the inhibition of the autophagy pathway.

Notably, we identified genes related to changes in erythrocyte differentiation that have not been well explored in previous studies. Compared to critical survivors, the non-survivors showed obvious activation of the erythrocyte differentiation pathway. Erythrocyte differentiation occurs in bone marrow, not peripheral blood. RBCs are reported to contain 8400 human genes, including erythrocyte differentiation genes [12]. The detected mRNAs in this study may be synthesized from previous processes of differentiations. Our results showed that dynamic changes in RBCs, rather than their overall level, may be an essential indicator of critical COVID-19 patient prognosis.

This study has several limitations. Firstly, sample size is a major limitation for the reliability of our statistical tests. Expanding the research population would increase the robustness of our conclusions. Another limitation is that the body mass index (BMI) information was incomplete in the current study, which may impact results as obesity is considered a strong predictor for poor prognosis [13, 14]. Moreover, other potential factors could account for the differences in critical non-survivors and survivors. For example, the sampling interval was not unified across patients, so some crucial physiological and biochemical changes may have been missed in our study. In addition, the time from onset to sampling was not consistent in each patient. Different times (7–11 days) are considered as the demarcation line between the early and late phases of the disease [15–18]. In our study, the earliest sampling date was 15 days after disease onset, resulting in a lack of samples collected during the early stage. Furthermore, in this study, age was significantly different between severe patients and critical non-survivors, consistent with the fact that older people exhibit a significantly higher fatality rate [19]. Although we adjusted for the effects of age in analysis, the significant differences may still impact our results, and may explain why the trend in RBC decline differed slightly between the validation and experimental groups (Fig. 3c). Nevertheless, as we collected multiple samples at different times in each patient with disease progression and validated the RBC and Hb results from a larger cohort, we believe that our results are consistent with real dynamic changes in COVID-19 patients.

In summary, we identified many different characteristics between critical COVID-19 survivors and non-survivors by leveraging a multi-omics view on longitudinally collected samples. Notably, transcriptional transition may be associated with the mortality and survival in critically ill COVID-19 patients. As critical non-survivors showed elevated erythrocyte differentiation and decreased RBC, erythrocyte biomarkers may be a promising and sensitive approach for predicting patient prognosis, which has not been well explored in previous studies. Furthermore, the protein–metabolite–lipid network suggested that activation of tryptophan metabolism and melatonin function disorder may prompt metabolism impairment in patients with different outcomes.

## Materials and methods

### Ethics statement

The Institutional Review Board of Tongji Hospital, Tongji Medical College, Huazhong University of Science and Technology approved the study (TJ-IRB20200405). Informed consents were obtained from patients or their family members. The rest of blood samples using for standard diagnostic tests were collected, posing no extra burden to patients.

### Patient enrollment

Blood samples from 33 COVID-19 patients without any complications were collected at Tongji Hospital of Huazhong University of Science and Technology from 19th February 2020 to 17th March 2020. A flowchart of sample preprocessing for this study is shown in Fig. 1a. Demographics and baseline characteristics of COVID-19 patients are shown in Additional file 1: Table S1. The mean age of the COVID-19 patients was 50.0 years old (standard deviation = 11.9), and the male:female ratio is 1.06:1. All patients were diagnosed in accordance with the guidelines for COVID-19 diagnosis and treatment (Trial Version 7) released by the National Health Commission of the People’s Republic of China. Selected patients were classified into two groups, including severely and critically ill. The critical state was defined with at least one of the following criteria: shock; acute respiratory distress syndrome (ARDS) requiring mechanical ventilation; and other organ dysfunction requiring admission to ICU. The severe state was defined with at least one of the following criteria: respiratory rate ≥ 30 times/min; arterial partial pressure of oxygen (PaO_2_)/fraction of inspired oxygen (FiO_2_) ≤ 300 mmHg; oxygen saturation ≤ 93% at resting state; and pulmonary imaging inspection showing significant injury progression by > 50% within 24–48 h. The definition of disease severity was consistent with previous study [20]. The exclusion criteria of comorbidities included hypertension, coronary heart disease, diabetes, chronic obstructive pulmonary disease, malignancy, surgical history, chronic kidney disease, cerebrovascular disease, immunodeficiency disease, chronic hepatitis, and tuberculosis.

### Sample preparation and nucleic acid extraction

Blood sample collection, blood cell separation, blood cell preservation, and serum preservation followed previous study [21]. After standard diagnostic tests, all anticoagulated venous blood samples treated with ethylenediaminetetraacetic acid disodium salt (EDTA-2Na) were separated by centrifugation at 3000 rpm, then rested at room temperature for 7 min. Whole blood cells were storage at − 80 °C. Before stored at − 80 °C, 200-μL aliquot of serum mixing with 800 μL of ice-cold methanol and a 200-μL aliquot of serum mixing with 800 μL of ice-cold isopropanol were prepared.

We isolated mRNA from whole blood cells of each sample using a QIAGEN miRNeasy Mini Kit (217004, Qiagen) following the manufacturer’s instructions. RNA extraction of all samples was performed in a Biosafety III Laboratory with Level III protection.

### Sequencing library construction and data generation

The mRNA library construction was performed as previously reported [21]. In total, 113 transcriptome profiles were generated as follows: (1) rRNA was removed using RNase H; (2) globin RNA was removed using a QAIseq FastSelect RNA Removal Kit; (3) cDNA fragments purified by magnetic beads were successively mixed with End Repair Mix, and A-Tailing Mix by pipetting, followed by incubation; (4) PCR amplification; (5) library quality control and pooling cyclization were conducted. MiRNAs were enriched and purified, followed by adaptor ligation, unique molecular identifier (UMI)-labeled primer addition, and reverse transcription.

Transcriptome mRNA libraries were sequenced by MGI2000 PE100 platform with 100-bp paired-end reads, while small RNA libraries were sequenced by BGI500 platform with 50-bp single-end reads resulting in at least 20M reads for each library.

### Proteomic analysis

Sera samples were processed as described previously [21] using data-independent acquisition [22] strategy based on UltiMate 3000 UHPLC liquid chromatography (Thermo Scientific, San Jose, USA) and Q Exactive HF mass spectrometer (Thermo Scientific, San Jose, USA). Spectronaut software was utilized to analyze the raw data obtained from 85 samples with default settings.

### Metabolomic analysis

In total, 100 μL of serum from each sample was analyzed using a spectrometer, LC following previous procedures [21]. Raw metabolite data obtained from 85 samples were analyzed using Compound Discoverer v3.1 (Thermo Fisher Scientific, USA).

### Lipidomic analysis

In brief, 100 μL of serum was analyzed using a Q Exactive mass spectrometer (Thermo Scientific, San Jose, USA) coupled with a Waters 2D UPLC (Waters, USA). Lipidsearch Version 4.1 (Thermo Fisher Scientific, USA) was employed to analyze the raw lipidomic data obtained from 85 samples.

### Gene expression quantitation, differential expression analysis and clustering

In total, we sequenced 113 mRNA samples. Transcriptome raw reads filtration was performed by SOAPnuke to remove low-quality reads (reads with low-quality base ratio (base quality < 5) > 20% and unknown base ('N' base) ratio > 5%). Reads were aligned to rRNA using Bowtie2 (v2.2.5) [23, 24]. Clean reads were mapped to the human reference genome using HISAT2 [25]. Gene expression (FPKM) was quantified by RSEM [26]. Genes with FPKM > 0.1 in at least one sample were retained.

DEseq2 (v1.4.5) was used to analyze differential mRNA expression by setting sex and age as confounders. Genes with a Benjamini–Hochberg *p-adjust* value < 0.05 were defined as significantly differentially expressed. Functional enrichment analyses, including GO term and KEGG pathway, were performed using Metascape with default parameters (*p*-value cutoff: 0.01; min enrichment: 1.5; pathway: GO Biological Process and KEGG Pathway) [27].

Principal component analysis (PCA) was performed to visualize transcriptome profiles and the unsupervised *k*-means clustering algorithm was used to cluster mRNA expression profiles, setting the initial cluster centers as 3, using the ade4 and stats R package.

Genes with an absolute value of log2FC greater than 1 between Cluster 1 and Cluster 2 were clustered using R package Mfuzz after log2-transformation [28]. The minimum centroid distance (MCD) for a range of cluster numbers for estimation of optimized number of clusters was calculated. The MCD elbow was used to determine final number of clusters. Gene clusters that changed markedly from Cluster 1 to Cluster 2 in each individual were selected and intersected to identify genes that changed consistently in all patients with state transitions.

### Estimation of cell proportion from bulk mRNA profiles

Cell heterogeneity and abundance were estimated from bulk mRNA profiles using CIBERSORTx and xCell [29, 30]. Cell types identified in more than half of samples were retained. Then, cell types that were significantly different in different status were retained.

### Differential expression of proteins, metabolites, and lipids

In total, 85 expression data were log2 transformed and analyzed using limma [31] after removing confounding factors of sex and age. The *P*-values were adjusted using Benjamini and Hochberg. Significantly differentially expressed proteins, metabolites or lipids were defined using the criteria of *p-adjust* value < 0.05.

### Heatmap expression profiles generation

All expression profiles, including mRNA, protein, metabolite, and lipid, removed confounders of sex and age using removeBatchEffect in R package limma. Processed expression matrix was used to plot a heatmap with the R package pheatmap.

### Network construction based on differential co-expression

To decipher dysregulated pathways in COVID-19 patients, a systems analysis framework based on a differential co-expression (DC) network was constructed. Firstly, the R package DGCA was recruited to calculate differences in correlation between molecular (including protein, lipid, and metabolite) pairs in various conditions (non-ICU vs ICU and Cluster 1 vs Cluster 2) [32]. The Spearman method was used to evaluate the correlation between each molecular pair, with only significantly differential correlations kept (*p* < 0.05). Three multiscale embedded networks were constructed using MEGENA for ICU comparison group, Cluster comparison group and identical differential correlation between them. Furthermore, to find highly connected molecules, which may have the same biological function, MEGENA was employed to detect their clustering structures in each network [33]. Finally, three networks, capturing significant differential correlation in each comparison group were retained: DC network between ICU and non-ICU (2167 nodes, 4801 edges), DC network between Cluster 1 and Cluster 2 (2167 nodes, 5818 edges), and identical DC network (1917 nodes, 2364 edges). Nodes with the highest degree in each network and molecule in the same module with nodes were explored to identify primary molecule changes. As melatonin and 5-hydroxyindole-3-acetic acid shared the same metabolic pathway, their subnetworks were integrated in the identical DC network. Network visualization was executed using Cytoscape [34].

## Supplementary Information

**Additional file 1: Table S1.** Baseline characteristics clinical parameters of recruited COVID-19 patients.

**Additional file 2: Table S2.** RBC and Hb characteristics of participants in discovery and validation groups.

**Additional file 3: Figure S1.** Heatmap of differential expression between the severe and critical groups in our transcriptome (A), proteome (B), lipidome (C), and metabolome (D) results.

**Additional file 4: Figure S2.** (A) Heatmap of differential expressed genes (logFC > 1) between Cluster 1 and Cluster 2. Individual effect and sampling day are considered as confounding factors. (B) Soft clustering of longitudinal gene dynamics in six patients containing Cluster 1-to-2 transition (FDR < 0.05) using differentially expressed genes in Figure S2A. Each patient is individually analyzed by using Mfuzz. The X-axis represents the sampling time. (C) Validation of differentially expressed gene from Cluster 1-to-2 by using Cluster 2-to-1 transcriptomic data. Color represents the patient’s ID. 99.4% up-regulated genes and 99.6% down-regulated genes exhibited reversed expression levels. (D) GO terms and KEGG pathways for differentially expressed genes between Cluster 1 and Cluster 2. The top 5 terms and pathways are represented. The size of dots denotes the − log10 of the *p-value*, the color denotes the expression levels.

**Additional file 5: Figure S3.** The comparison of expression levels of 5-hydroxyindole-3-acetic (A) and l-kynurenine (B) between Cluster 1 and Cluster 2.

## Data Availability

Data for this project will be available upon request.

## Code availability

Custom scripts for data analysis in this study were present in https://github.com/Shiywa/COVID-19-time-series/tree/master.
