# Supplementary material for: Longitudinal multi-omics transition associated with fatality in critically ill COVID-19 patients
Source: Intensive Care Med Exp. 2021 Mar 15;9:13. doi: 10.1186/s40635-021-00373-z (PMC7957447; doi:10.1186/s40635-021-00373-z)
Supplement: Supplementary file 1 — Additional file 1: Table S1. Baseline characteristics clinical parameters of recruited COVID-19 patients. [file 40635_2021_373_MOESM1_ESM.docx]

**TableS1. Baseline Characteristics Clinical Parameters of Recruited COVID-19 Patients.**

| **Variables** | **Severe (n=18)** | **Critical Survivors (n=4)** | **Critical (non-survivors) (n=11)** | **Total (n=33)** | **P** **value1** | **P value2** |
| --- | --- | --- | --- | --- | --- | --- |
| **Sex, NO.(%)** |  |  |  |  | 0.28 | 0.33 |
| Male | 10 (55.6) | 3 (75) | 4 (36.4) | 17 (51.5) | ----- | ----- |
| Female | 8 (44.4) | 1 (25) | 7 (63.6) | 16 (48.5) | ----- | ----- |
|  |  |  |  |  |  |  |
| **Age** |  |  |  |  | 0.12 | 8.2E-5 |
| Mean ± SD | 42.4±7.5 | 50.3±12.8 | 62.2±6.4 | 50.0±11.9 | ----- | ----- |
| Median [IQR] | 46 (40.25-48) | 45 (42-53.25) | 63 (58-67.5) | 48 (42-59) | ----- | ----- |
| Range | 25-49 | 42-69 | 49-70 | 25-70 | ----- | ----- |
|  |  |  |  |  |  |  |
| **Time from Onset to Admission, Days** |  |  |  |  |  |  |
| Mean ± SD | 12.06±6.37 | 32±17.11 | 12.64±7 | 14.67±10.35 |  |  |
| Median [IQR] | 12.5 (6.25-15) | 38.5 (28-42.5) | 11 (9.5-14.5) | 13 (7-16) |  |  |
| Range | 4-24 | 7-44 | 3-30 | 3-44 |  |  |
|  |  |  |  |  |  |  |
| **Symptoms, NO.(%)** |  |  |  |  |  |  |
| Fever | 17 (94.44) | 4 (100) | 8 (72.73) | 29 (87.88) | 0.52 | 0.16 |
| Fatigue | 2 (11.11) | 1 (25) | 3 (27.27) | 6 (18.18) | 1 | 0.44 |
| Dry cough | 12 (66.67) | 1 (25) | 9 (81.82) | 22 (66.67) | 0.08 | 0.16 |
| Expectoration | 7 (38.89) | 0 (0) | 7 (63.64) | 14 (42.42) | 0.08 | 0.08 |
| Myalgia | 1 (5.56) | 0 (0) | 0 (0) | 1 (3.03) | 1 | 1 |
| Dizziness | 0 (0) | 0 (0) | 2 (18.18) | 2 (6.06) | 1 | 0.2 |
| Cephalalgia | 3 (16.67) | 0 (0) | 1 (9.09) | 4 (12.12) | 1 | 1 |
| Abdominal pain | 1 (5.56) | 0 (0) | 0 (0) | 1 (3.03) | 1 | 1 |
| Chest pain | 0 (0) | 0 (0) | 2 (18.18) | 2 (6.06) | 1 | 0.2 |
| Diarrhea | 5 (27.78) | 0 (0) | 5 (45.45) | 10 (30.3) | 0.23 | 0.26 |
| Vomiting | 1 (5.56) | 0 (0) | 0 (0) | 1 (3.03) | 1 | 1 |
| Dyspnea | 8 (44.44) | 0 (0) | 9 (81.82) | 17 (51.52) | 0.01 | 0.01 |
|  |  |  |  |  |  |  |
| **Treatment, NO.(%)** |  |  |  |  |  |  |
| Oxygen inhalation | 18 (100) | 4 (100) | 10 (90.91) | 32 (96.97) | 1 | 0.45 |
| Antibiotic treatment | 17 (94.44) | 4 (100) | 10 (90.91) | 31 (93.94) | 1 | 1 |
| Antiviral therapy | 17 (94.44) | 4 (100) | 10 (90.91) | 31 (93.94) | 1 | 1 |
| Immunization therapy | 10 (55.56) | 3 (75) | 8 (72.73) | 21 (63.64) | 1 | 0.59 |
| Glucocorticoid therapy | 11 (61.11) | 4 (100) | 8 (72.73) | 23 (69.7) | 0.52 | 0.32 |
| Traditional Chinese Medicine | 18 (100) | 4 (100) | 8 (72.73) | 30 (90.91) | 0.52 | 0.06 |
| CKRT | 0 (0) | 3 (75) | 5 (45.45) | 8 (24.24) | 0.57 | 0 |
| ECMO | 0 (0) | 2 (50) | 0 (0) | 2 (6.06) | 0.06 | 0.01 |
| IMV | 0 (0) | 2 (50) | 10 (90.91) | 12 (36.36) | 0.15 | 0 |
|  |  |  |  |  |  |  |
| **Clinical Parameters (normal range)** | |  |  |  |  |  |
| CRP (mg/L, <1mg/L, median, [IQR]) | 2.1 (1.2-7.1) | 95.3 (40.85-125.8) | 99.3 (67.75-213.25) | 69.4 (10.5-134.95) | 0.31 | 3.00E-08 |
| WBC (×10⁹/L, 3.5-9.5, median, [IQR]) | 6.76 (5.42-7.51) | 12.25 (10.55-14.14) | 10.92 (8.71-14.66) | 9.79 (6.55-13.02) | 8.9E-7 | 2.20E-12 |
| Lymph (×10⁹/L, 1.1–3.2, median, [IQR]) | 1.63 (1.37-1.99) | 1.42 (0.98-1.74) | 0.52 (0.26-0.83) | 1.14 (0.52-1.73) | 0.43 | 4.80E-11 |
| NEU (×10⁹/L, 1.8-6.3, median, [IQR]) | 4.15 (3.09-5.2) | 9.61 (7.5-10.99) | 10.14 (7.47-13.04) | 7.74 (4.31-10.84) | 0.077 | 8.10E-09 |
| PLT (×10⁹/L, 125–350, median, [IQR]) | 217 (171-260) | 136 (122-185) | 130 (71.5-170.5) | 159 (116.5-220) | 0.062 | 2.50E-16 |
| Hb (g/L, 115-150, median, [IQR]) | 131 (122-140) | 82 (74.5-92.5) | 90 (80-104.5) | 100 (83.5-128) | 0.37 | 0.075 |
| ALT (U/L, ≤41, median, [IQR]) | 47.5 (22.75-80.75) | 40.5 (21-58.5) | 28 (20-43.25) | 36 (21-55.5) | 0.21 | 0.48 |
| AST (U/L, ≤40, median, [IQR]) | 34 (18.75-54.5) | 24.5 (20-40.25) | 33 (22-47) | 32.5 (20.75-47.25) | 0.65 | 0.77 |
| TBIL (mmol/L, ≤26, median, [IQR]) | 11 (7.05-13.95) | 8 (7.62-19.2) | 11.5 (6.95-17.3) | 10.2 (7-16.75) | 0.046 | 0.051 |
| ALB (g/L, 35-52, median, [IQR]) | 37.45 (35.75-41.23) | 39.3 (32.9-42.05) | 32.3 (28.65-40.55) | 36.6 (29.95-41.25) | 0.75 | 0.25 |
| GLO (g/L, 20-35, median, [IQR]) | 28.1 (25.98-31.27) | 30.9 (26.78-34.65) | 29.3 (27.25-32.95) | 29.1 (26.65-32.95) | 0.096 | 1.80E-12 |
| IL.6 (pg/mL, <7, median, [IQR]) | 2.29 (1.5-4.06) | 75.12 (33.27-125.47) | 85.48 (31-207.1) | 52.26 (7.46-134.6) | 0.4 | 2.3E-08 |
| IL.10 (pg/mL, <9.1, median, [IQR]) | 5 (5-5) | 5.3 (5-9.57) | 7.5 (5-16.2) | 5 (5-10) | 0.22 | 0.0024 |
| IL.8 (pg/mL, <62, median, [IQR]) | 5 (5-5) | 23.15 (19.45-28.35) | 37.4 (26.6-108) | 23.7 (12.3-43.8) | 0.0034 | 7.8E-7 |
| TNF.α (pg/mL, <8.1, median, [IQR]) | 6.7 (4-10.18) | 19.45 (13.53-23.78) | 7.75 (5.22-14.12) | 9.95 (5.4-18.65) | 0.0082 | 0.00052 |
| IL-1β (pg/mL, <5pg/mL, median, [IQR]) | 5 (5-5) | 5 (5-5.15) | 5 (5-5) | 5 (5-5) | 0.43 | 0.54 |
| IL.2R (U/mL, 223-710, median, [IQR]) | 296 (229-534) | 1531.5 (1128.5-1811.25) | 1070 (399-1548) | 849.5 (374.75-1536) | 0.053 | 4.3E-6 |
| ESR (mm/h, 0–15, median, [IQR]) | 20 (7.5-37) | 32.5 (18.5-43) | 31 (22-71) | 28 (14.25-42.5) | 0.84 | 0.055 |
| PCT (ng/mL, <0.5, median, [IQR]) | 0.03 (0.03-0.06) | 1.05 (0.5-2.46) | 0.54 (0.2-1.06) | 0.39 (0.06-1.02) | 0.03 | 7.20E-12 |
| D-dimer (μg/mL, <0.5, median, [IQR]) | 0.3 (0.22-0.74) | 7.77 (3.82-20.37) | 3.67 (2.22-7.27) | 3.36 (0.76-7.46) | 0.0091 | 5.70E-12 |

|  |  |  |  |  |
| --- | --- | --- | --- | --- |

Abbreviations: IQR, interquartile range; SD, Standard Deviation; CRP, C-reactive protein; WBC, White blood cell; Lymph, Lymphocyte; NEU, Neutrophil; PLT, Platelet; Hb, Haemogobin; ALT, Alanine aminotransferase; AST, Aspartate aminotransferase; TBIL, Total bilirubin; ALB, Albumin; GLO, Globin; IL, interleukin; TNF, tumor necrosis factor; ESR, Erythrocyte sedimentation rate; PCT, Procalcitonin; CKRT, continuous kidney replacement therapy; ECMO, Extracorporeal Membrane Oxygenation; IMV, invasive mechanical ventilation

P value1: significant test result between critical survivors and non-survivors; P value 2: significant test result of three groups.
