# Supplementary material for: Longitudinal multi-omics transition associated with fatality in critically ill COVID-19 patients
Source: Intensive Care Med Exp. 2021 Mar 15;9:13. doi: 10.1186/s40635-021-00373-z (PMC7957447; doi:10.1186/s40635-021-00373-z)
Supplement: Supplementary file 2 — Additional file 2: Table S2. RBC and Hb characteristics of participants in discovery and validation groups. [file 40635_2021_373_MOESM2_ESM.docx]

**TableS2. RBC and Hb Characteristics of Participants in Discovery and Validation Groups.**

| **Variables** | **Discovery** | | **Validation** | |
| --- | --- | --- | --- | --- |
|  | **Critical(Survival)(n=4)** | **Critical(Death)(n=11)** | **Critical(Survival)(n=51)** | **Critical(Death)(n=63)** |
| **Sex, NO.(%)** |  |  |  |  |
| Male | 3(75) | 4(36.4) | 23(45.1) | 37(58.7) |
| Female | 1(25) | 7(63.6) | 28(54.9) | 26(41.3) |
|  |  |  |  |  |
| **Age** |  |  |  |  |
| Mean ± SD | 50.3±12.8 | 62.2±6.4 | 60.18±11.88 | 66.52±13.08 |
| Median [IQR] | 45(42-53.25) | 63(58-67.5) | 61(51.5-68.5) | 68(60-75) |
| Range | 42-69 | 49-70 | 36-82 | 24-92 |
|  |  |  |  |  |
| **Clinical parameters (normal range)** | |  |  |  |
| RBC (×10^12^/L, 4.0-5.5, median, [IQR]) | 2.73(2.55-2.95) | 3.34(2.81-3.97) | 3.91(3.56-4.36) | 3.38(2.64-3.95) |
| Hb (g/L, 115-150, median, [IQR]) | 86(81-91) | 100(83.5-118) | 105(82-123) | 120(110-133) |

Abbreviations: IQR, interquartile range; SD, Standard Deviation; RBC, red blood cell; Hb, Haemogobin.
