# Supplementary figures and images for: Longitudinal multi-omics transition associated with fatality in critically ill COVID-19 patients
Source: Intensive Care Med Exp. 2021 Mar 15;9:13. doi: 10.1186/s40635-021-00373-z (PMC7957447; doi:10.1186/s40635-021-00373-z)

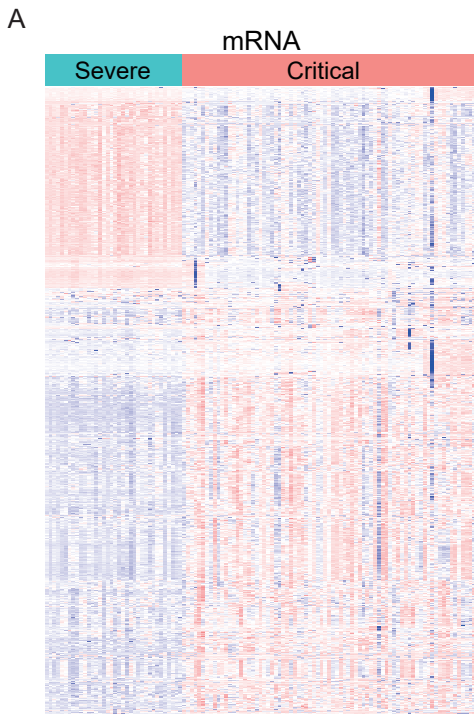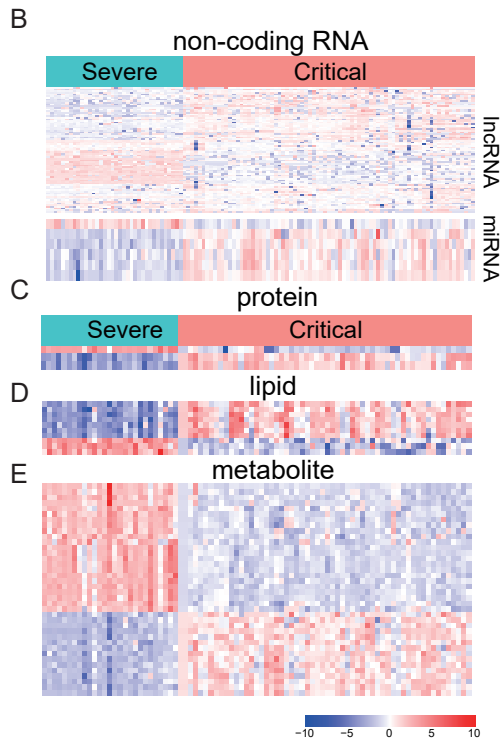

Supplement: Supplementary file 3 — Additional file 3: Figure S1. Heatmap of differential expression between the severe and critical groups in our transcriptome (A), proteome (B), lipidome (C), and metabolome (D) results. [file 40635_2021_373_MOESM3_ESM.pdf]

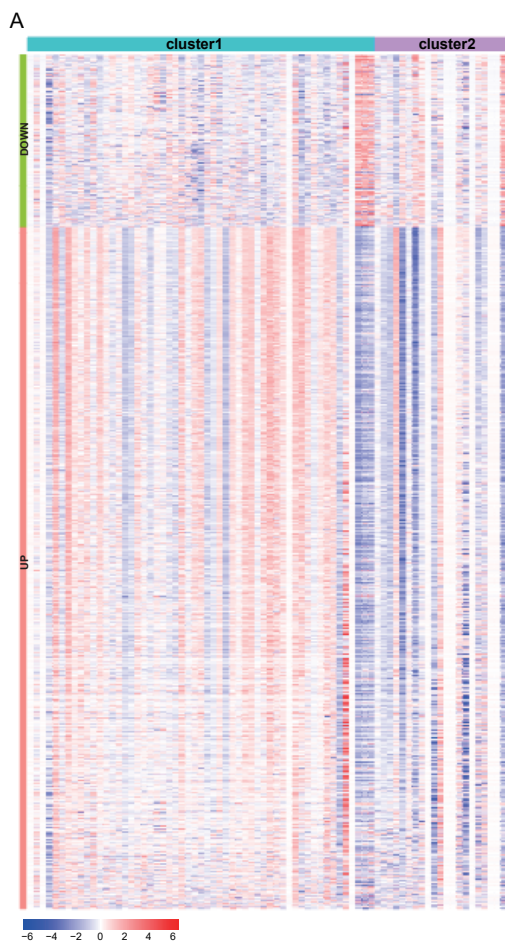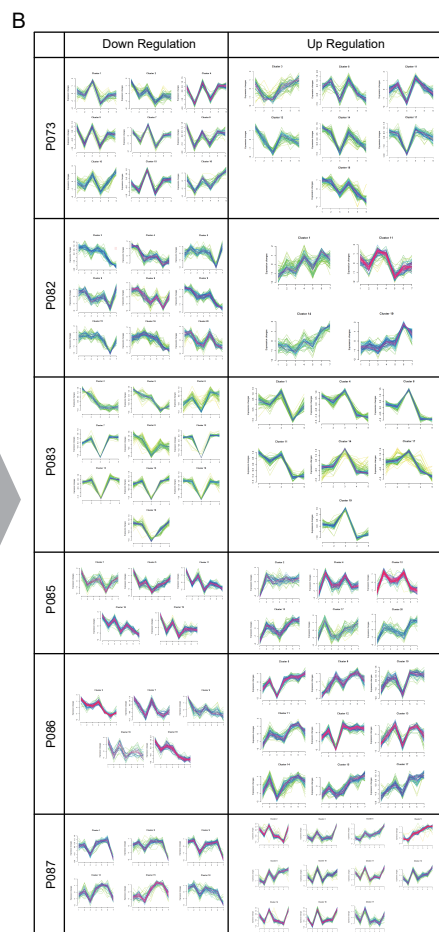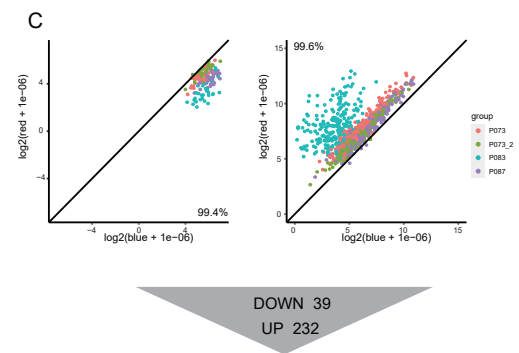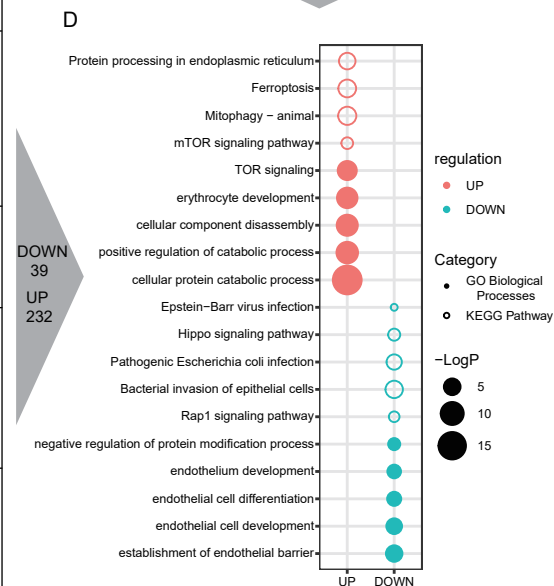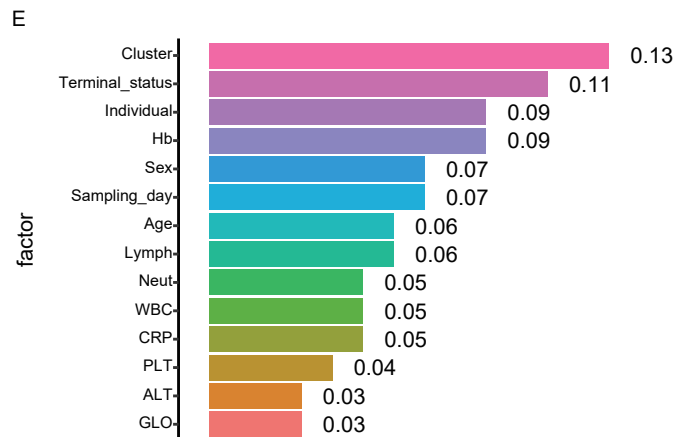

Supplement: Supplementary file 4 — Additional file 4: Figure S2. (A) Heatmap of differential expressed genes (logFC > 1) between Cluster 1 and Cluster 2. Individual effect and sampling day are considered as confounding factors. (B) Soft clustering of longitudinal gene dynamics in six patients containing Cluster 1-to-2 transition (FDR < 0.05) using differentially expressed genes in Figure S2A. Each patient is individually analyzed by using Mfuzz. The X-axis represents the sampling time. (C) Validation of differentially expressed gene from Cluster 1-to-2 by using Cluster 2-to-1 transcriptomic data. Color represents the patient’s ID. 99.4% up-regulated genes and 99.6% down-regulated genes exhibited reversed expression levels. (D) GO terms and KEGG pathways for differentially expressed genes between Cluster 1 and Cluster 2. The top 5 terms and pathways are represented. The size of dots denotes the − log10 of the p-value, the color denotes the expression levels. [file 40635_2021_373_MOESM4_ESM.pdf]

A

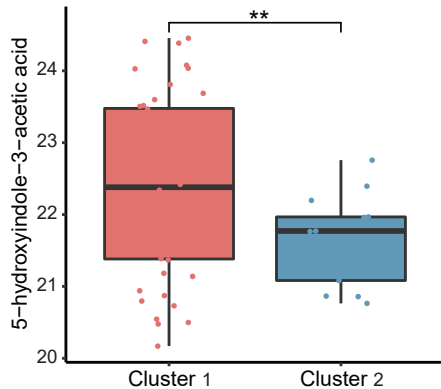

B

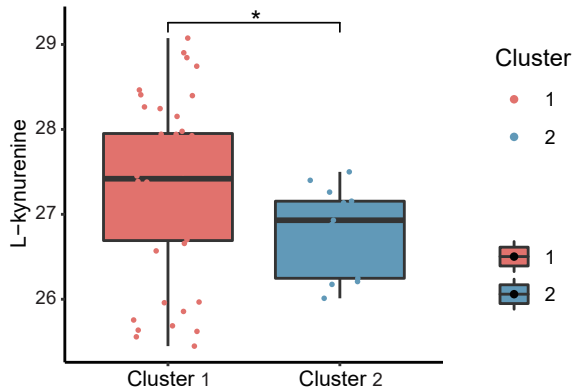

Supplement: Supplementary file 5 — Additional file 5: Figure S3. The comparison of expression levels of 5-hydroxyindole-3-acetic (A) and l-kynurenine (B) between Cluster 1 and Cluster 2. [file 40635_2021_373_MOESM5_ESM.pdf]
